# The disruptive role of LRG1 on the vasculature and perivascular microenvironment

**DOI:** 10.3389/fcvm.2024.1386177

**Published:** 2024-04-30

**Authors:** Athina Dritsoula, Carlotta Camilli, Stephen E. Moss, John Greenwood

**Affiliations:** UCL Institute of Ophthalmology, University College London, London, United Kingdom

**Keywords:** LRG1, TGF-β, vascular dysfunction, inflammation, fibrosis, angiogenesis, vessel normalization, vascular

## Abstract

The establishment of new blood vessels, and their subsequent stabilization, is a critical process that facilitates tissue growth and organ development. Once established, vessels need to diversify to meet the specific needs of the local tissue and to maintain homeostasis. These processes are tightly regulated and fundamental to normal vessel and tissue function. The mechanisms that orchestrate angiogenesis and vessel maturation have been widely studied, with signaling crosstalk between endothelium and perivascular cells being identified as an essential component. In disease, however, new vessels develop abnormally, and existing vessels lose their specialization and function, which invariably contributes to disease progression. Despite considerable research into the vasculopathic mechanisms in disease, our knowledge remains incomplete. Accordingly, the identification of angiocrine and angiopathic molecules secreted by cells within the vascular microenvironment, and their effect on vessel behaviour, remains a major research objective. Over the last decade the secreted glycoprotein leucine-rich α-2 glycoprotein 1 (LRG1), has emerged as a significant vasculopathic molecule, stimulating defective angiogenesis, and destabilizing the existing vasculature mainly, but not uniquely, by altering both canonical and non-canonical TGF-β signaling in a highly cell and context dependent manner. Whilst LRG1 does not possess any overt homeostatic role in vessel development and maintenance, growing evidence provides a compelling case for LRG1 playing a pleiotropic role in disrupting the vasculature in many disease settings. Thus, LRG1 has now been reported to damage vessels in various disorders including cancer, diabetes, chronic kidney disease, ocular disease, and lung disease and the signaling processes that drive this dysfunction are being defined. Moreover, therapeutic targeting of LRG1 has been widely proposed to re-establish a quiescent endothelium and normalized vasculature. In this review, we consider the current status of our understanding of the role of LRG1 in vascular pathology, and its potential as a therapeutic target.

## Introduction

The formation of a vascular network is a fundamental prerequisite in serving the needs of the surrounding tissue and enabling normal tissue function. The process of angiogenesis, whether during development or postnatally as during reproduction and wound healing, has been studied extensively and many of the controlling elements have been defined (1, 2). Vascular endothelial growth factor (VEGF) has emerged as the master regulator, but other factors also contribute to vessel growth and the subsequent stabilization and maturation processes, which are necessary for the establishment of functional vessels (3). The vasculature, however, is highly heterogeneous with vessel structure and function depending on position in the vascular hierarchy and on the physiological requirements of the surrounding tissue. To achieve such diverse functionality, different local signaling factors are required during development and throughout life to maintain this site-specific specialization. Nevertheless, some ubiquitous signaling interactions are deemed essential for vessel maturation, most notably the crosstalk between the endothelium and perivascular mural cells, which are critical to vascular homeostasis. Indeed, disruption of this cellular interplay is recognised as a major factor in the destabilization of existing vessels in several diseases.

Aside from the disruption of existing vessels, the formation of new vessels in disease has attracted enormous attention as they are usually abnormal, often forming a chaotic and immature network that may be poorly perfused, leaky, and fragile (2). Whilst many of the factors driving new vessel growth in disease are also those responsible for developmental and physiological angiogenesis, it is clear that differences must exist. Thus, it has been proposed that the balance of expression between pro- and anti-angiogenic factors may be disturbed, while additional disease-specific players may corrupt the normal angiogenic and maturation processes. Although our understanding of these factors, and the associated signaling pathways that disrupt both new and existing vascular structure and function is considerable, there remain significant gaps in our knowledge as highlighted by the large number of patients who fail to respond favourably to standard of care therapies aimed at alleviating vascular dysfunction (2, 4).

The interaction between the endothelium and perivascular mural cells is an imperative and extremely complex process that is central to normal blood vessel development and long-term homeostasis. Abnormalities in this relationship are implicated in numerous vascular diseases, such as diabetic retinopathy, pulmonary hypertension, kidney disease and cancer, where decreased mural cell coverage or abnormal recruitment results in destabilized leaky microvessels and vascular rarefication (5–9). In addition, activation or de-differentiation of vascular smooth muscle cells and pericytes can lead to vascular remodeling and medial thickening in larger vessels (5). Also emerging is the disruptive role played by endothelial to mesenchymal transition (EndMT) (10), a process similar to epithelial to mesenchymal transition (EMT), in which TGF-β plays a predominant role. Irrespective of cause, when vessels become dysfunctional, they not only fail to provide their selective barrier and delivery functions but can also negatively influence the behaviour of surrounding tissue. Designing therapeutic strategies, therefore, that aim to normalize the vasculature has received increasing attention, especially in cancer (11), but further insight into our understanding of interactions that drive vascular dysfunction in disease is needed. Through such improved insight, new therapeutic targets will be identified for which activation or inhibition will enable repair of dysfunctional vessels and the facilitation of physiological revascularization during disease. One recently identified candidate, the angiocrine factor leucine-rich α-2 glycoprotein 1 (LRG1), is emerging as a ubiquitous and potentially critical player in driving vascular pathology, and therefore offers substantial potential as a new therapeutic target in many different diseases. This review aims to present the diverse and emerging vasculopathic roles that LRG1 has on the vasculature and its influence on the local microenvironment.

## LRG1 in homeostasis and disease

**Figure 1:**
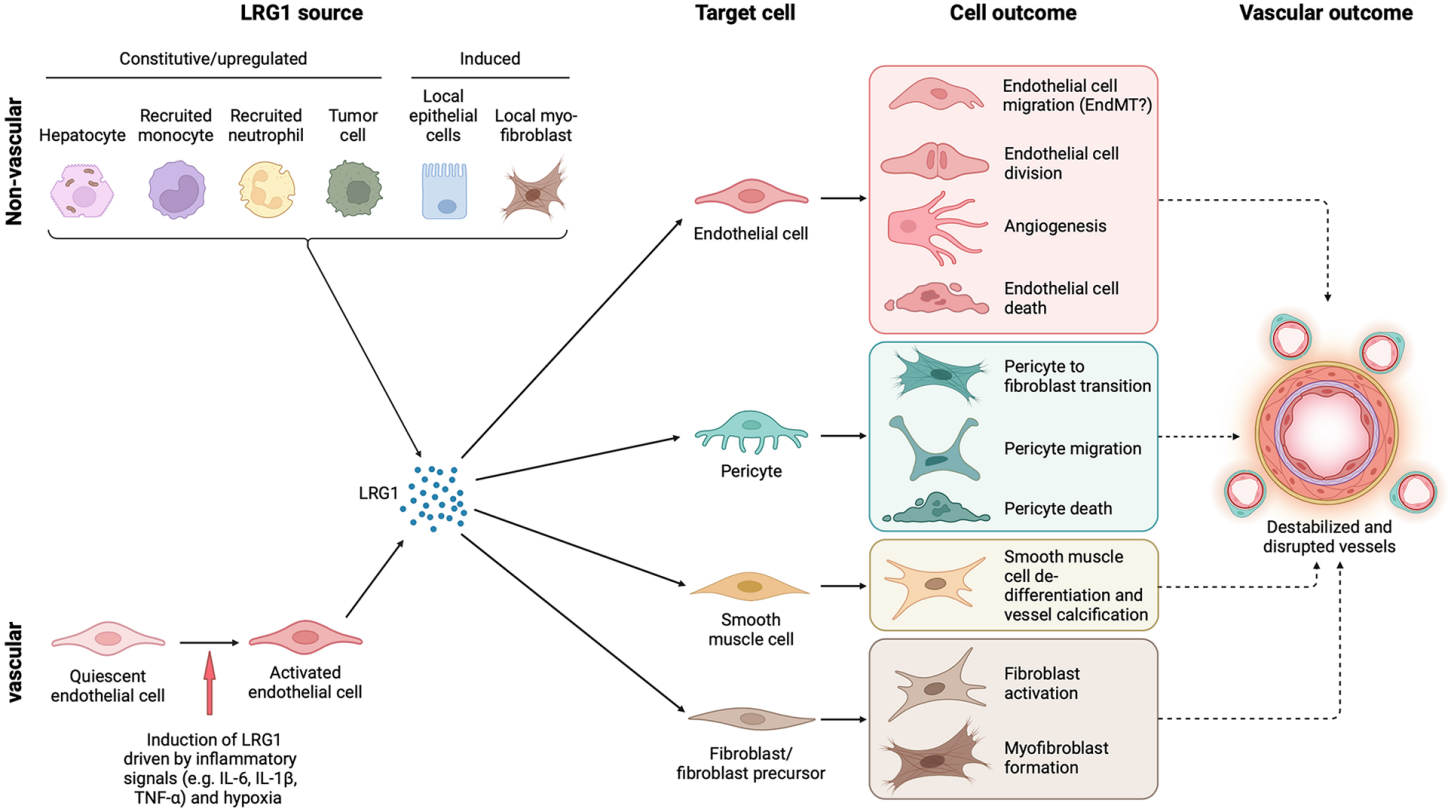
Cell source, target cells and outcome of LRG1 in the vasculature. LRG1 is expressed constitutively by only a few cell types, including hepatocytes and neutrophils, but during disease can be induced in various other cells. In the vasculature, endothelial cells are the only source of LRG1, but other local cells may also be induced to express LRG1 such as fibroblasts and tumor cells. Irrespective of the source, LRG1 affects the function of all cellular components of blood vessels resulting in vascular damage. Vascular dysfunction is closely associated with fibrosis, and both may influence each other through LRG1 signaling. EndMT, endothelial to mesenchymal transition. Created with BioRender.com.

LRG1 is a member of the conserved family of leucine-rich repeat glycoproteins that are involved in numerous physiological functions, including protein-protein interactions and innate immune responses (12, 13). Under normal conditions LRG1 is expressed almost exclusively by liver hepatocytes, from where it is secreted into the circulation and maintained at an approximate concentration of 20–50 μg/ml in the plasma (14) (Figure 1). Neutrophils, which are powerful mediators of innate immunity, are also known to express LRG1 constitutively and are likely to contribute to LRG1-mediated pathogenic effects in inflamed tissues and diseased vasculature (Figure 1). In these cells, LRG1 is stored within granules, and its expression has been associated with accelerated neutrophilic granulopoiesis, suggesting a potential role for LRG1 in myeloid cell differentiation (15, 16). Moreover, LRG1 has been shown to be involved in the formation of neutrophil extracellular traps (NETs), a process known as NETosis, via a signaling pathway involving ALK5 and AKT (17). NETosis is an innate immune response initiated against pathogens but is also frequently associated with vascular dysfunction (18). These findings, together with increased LRG1 levels in serum in response to microbial or viral infections and other inflammatory stimuli, as well as the high binding affinity of LRG1 for cytochrome c (Cyt c) following its release upon apoptotic signals, have led to the proposal that LRG1 functions as an acute phase protein involved in the innate immune response (19, 20). Aside from these major sources of LRG1, low levels of expression have been reported under normal conditions in other tissues including lung, kidney, heart, brain, testis, and the vasculature, where LRG1 is predominantly expressed in endothelial cells (21) (Figure 1). Many of these studies, however, use immunohistochemical detection, which might reflect the presence of extracellular, and matrix/endothelial cell sequestered LRG1 derived from the circulation. Nevertheless, a recent transcriptomics study dissecting the molecular heterogeneity of blood vascular endothelial cells from skin has shown that *LRG1* transcript is exclusively found in the blood vessels and more specifically in healthy skin enriched in the postcapillary venules (22).

Although current evidence suggests that LRG1 expression may be important to the functions of different organs and processes, its primary physiological role remains poorly defined, with *Lrg1*-deficient mice presenting with normal development and fertility, and no obvious phenotype indicating a non-essential role. In recent years, however, accumulating evidence points to the involvement of LRG1 in a wide range of diseases (23, 24). Since 2013, when LRG1 was initially identified as a key vasculopathic molecule in abnormal angiogenesis (25), more causative roles have been attributed. Among others, LRG1 has been found to drive IL-6-dependent pathological angiogenesis through the STAT3 signaling pathway (26, 27), destabilize tumor vasculature and promote tumor growth and metastasis (28–32), drive chronic kidney and lung disease (33–40), stimulate fibrosis (33, 34, 41–48), contribute to diabetes-related pathology (17, 49–57), and regulate pathological placental angiogenesis (58). High circulating LRG1 levels have been reported in many cancers, where LRG1 has been proposed, or used, as a prognostic and diagnostic marker. Indeed, high LRG1 levels in cancer patients' plasma correlate with poor prognosis and survival (59–61), and resistance to standard of care therapy (62). The involvement and roles of LRG1 in key vascular processes are the scope of this review and will be discussed in detail.

## Regulation of LRG1 expression

**Figure 2:**
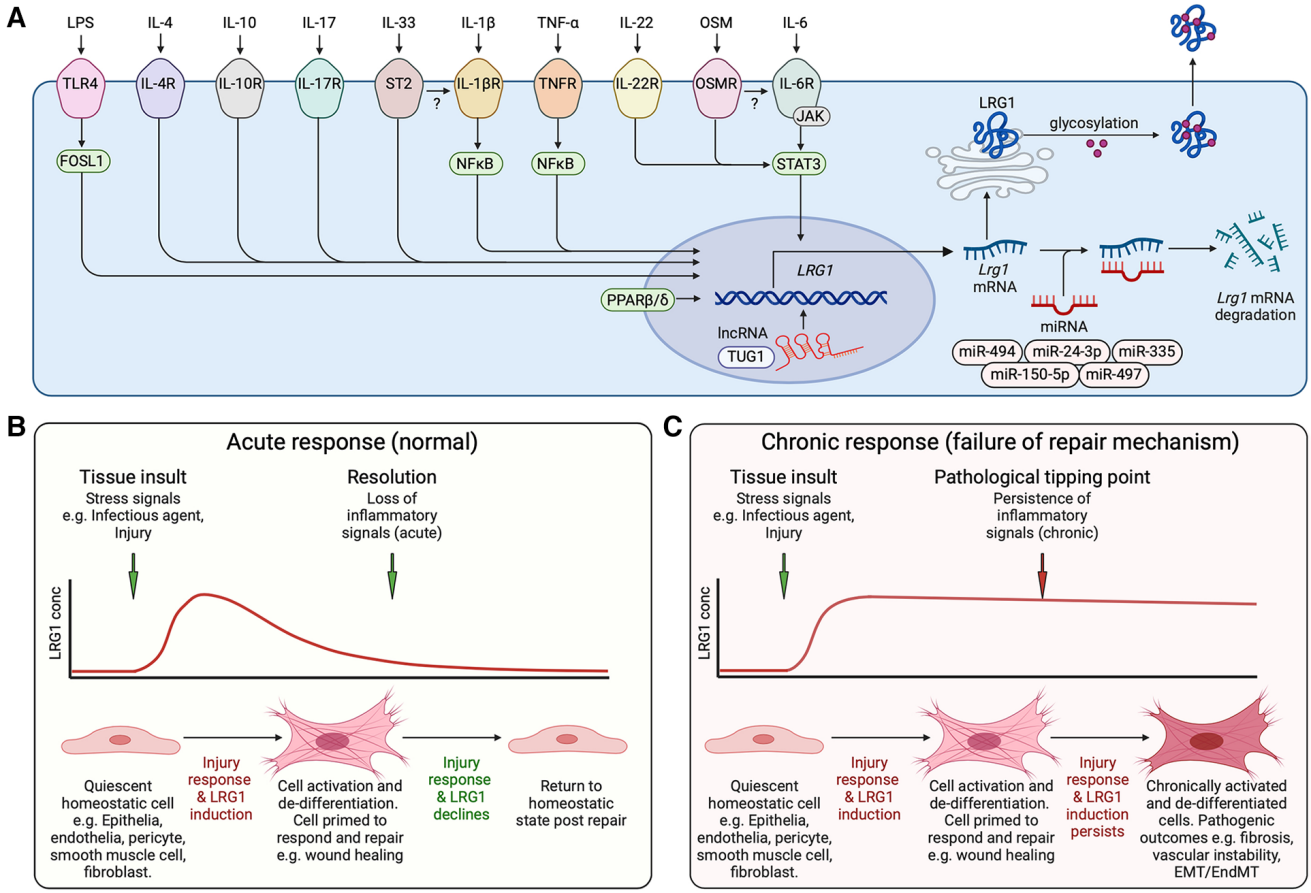
Induction of LRG1. (**A**) Schematic representation of inflammatory factors reported to activate LRG1 gene expression and known transcriptional and post-transcriptional regulatory mechanisms. Cytokines and lipopolysaccharide (LPS) acting through their cognate receptors and downstream transcription factors drive *LRG1* gene induction. lncRNA TUG1 facilitates *LRG1* transcription while miR-335, miR-494, miR-497, miR-150-5p and miR-24-3p promote the degradation of *LRG1* mRNA. LRG1 protein is differentially glycosylated in a cell- and function-specific manner that may affect its activity. The secreted protein may combine to form dimers and trimers. OSM, oncostatin M; IL, interleukin; TLR4, toll-like receptor 4; lncRNA, long non-coding RNA; miRNA, microRNA. (**B**) LRG1 is induced during cell damage and infection as part of the repair mechanism. In the presence of LRG1 and TGF-β, we propose that cells are activated and de-differentiated into a less mature state to enable wound healing. As inflammatory signals subside, LRG1 expression diminishes allowing resolution of tissue damage and cells to return to a quiescent state. (**C**) Under chronic conditions, inflammatory signals maintain LRG1 expression perpetuating the unstable cell state and preventing completion of the healing process. EMT, epithelial to mesenchymal transition; EndMT, endothelial to mesenchymal transition. Created with BioRender.com.

Several transcriptomic and post-transcriptional regulatory studies have shown that the IL-6/STAT3 signaling pathway is a major activator of LRG1 expression (26, 29, 31, 63). Indeed, it was recently reported that LRG1 transcriptional activation is abolished upon deletion of the STAT3 binding site on the *LRG1* promoter (26). Inflammation seems to be an important driver of LRG1 expression, with many different cytokines activating LRG1 in a range of cells and disease settings (Figure 2). These include IL-6, IL-1β, TNF-α, IL-17, IL-4, IL-10 and IL-33 and they may act either alone or synergistically (36, 64–68). In addition, PPARβ/δ is able to bind the *LRG1* promoter to activate transcription as reported in a chromatin immunoprecipitation assay (47), and similarly, both ELK1 and ELK4 transcription factors have been shown to initiate *LRG1* transcription upon mechanical strain (41) and through cooperation with Sp1/Sp3 complex (69), respectively. FOS-like 1 was also identified as a novel activator of *LRG1* in a transcriptomics study (39). There is also evidence that hypoxia can induce LRG1 expression (70), consistent with the presence of potentially active HIF-1α binding elements in the *LRG1* promoter. At the post-transcriptional and post-translational levels, microRNAs, long non-coding RNAs, and histone modifications have also been reported to regulate LRG1 expression (23) (Figure 2). Furthermore, different glycosylation patterns have been identified and these might influence LRG1 function (23). Overall, while experimental evidence that unravels the pleiotropic functions of LRG1 continues to accumulate, further work is required to establish the key regulatory mechanisms that control LRG1 expression and function, some of which will be discussed in detail later.

## LRG1 and angiogenesis

In the early stages of development, blood vessels are formed through a tightly orchestrated process called vasculogenesis, where mesoderm-derived angioblasts, the precursor of endothelial cells, establish angioblastic cords that advance into a primitive vascular plexus and subsequently into blood vessels. At later stages of development, and postnatally in the reproductive system and during physiological tissue repair, angiogenesis occurs in response to tissue growth and hypoxia. The angiogenic mechanism is tightly regulated and influenced by pro-angiogenic factors, with VEGF being a key master regulator, essential for the development and maintenance of the vascular system (3). Other growth factors with established pro-angiogenic activities include the FGFs, PDGF, angiopoietins, HGF and the TGF-β superfamily. In particular, PDGF and the angiopoietins exert their functions on the endothelium in a paracrine manner through their binding to, or their expression by, perivascular mural cells. In addition, angiopoietins regulate vascular homeostasis and promote vessel stability through the recruitment of mural cells and mediate their interactions with the endothelium (71). Among other potent angiogenic molecules are angiopoietin-like 4 (72), apelin (73), Frizzled A (74, 75), thrombomodulin (76), AGGF1 (77), Slit3 (78), as well as a plethora of pro-angiogenic peptides, the therapeutic potential of which is reviewed elsewhere (79). Aside from the established regulators of angiogenesis, there are many ancillary factors that influence vessel growth and maturation and which, under certain circumstances, may even circumvent the angiogenic dependency on VEGF. As will be seen in this review, LRG1 is now considered to be one of these adjunct pro-angiogenic factors and hence can be added to this extensive list. However, contrary to many of the factors mentioned above, LRG1 does not play a role in developmental or physiological angiogenesis as knock-out mice exhibit no overt phenotype, breed successfully and live a normal life span (21). In the context of disease, however, LRG1 is now recognised as a potent pro-neoangiogenic factor as it has been shown to promote pathological angiogenesis in numerous diseases and *de novo* growth in *in vitro* experimental settings (Table 1). It is worth noting, however, that angiogenesis under *in vitro*/*ex vivo* conditions is unlikely to represent normal angiogenesis, as the environment is more aligned to a pathogenic than homeostatic state. As outlined in more detail in the sections below, and with the caveat above, several studies have investigated the pro-angiogenic properties of LRG1 and have shown that it can act directly on endothelial cells, inducing cell proliferation and tube formation *in vitro* as well as on other cells impacting proliferation, migration and viability (17, 27, 34, 41, 80–84, 86). In addition, LRG1 promotes vessel growth and sprouting in *ex vivo* mouse metatarsal bone and aortic ring angiogenesis assays (25, 26). This is attenuated in explant tissues derived from *Lrg1*^−/−^ mice or when a function-blocking anti-LRG1 antibody is used (25, 26, 84, 94). Similarly, knocking down LRG1 through siRNA silencing decreased the tube formation capacity of HUVEC co-cultured with pancreatic cancer cells (81). Vessels that develop in response to LRG1 stimulation, in the absence of other exogenous drivers, exhibit a pathological phenotype. In particular, LRG1 treatment of metatarsal bones and aortic rings resulted in vessels with reduced coverage of αSMA^+^ and NG2^+^ mural cells (26). Although the mechanisms behind this remain unknown, it seems likely that both autocrine and paracrine mechanisms affect the recruitment, proliferation, migration and differentiation state of mural cells in a LRG1-dependent manner. These studies raise the question whether LRG1 is a true angiogenic factor or whether it affects the stability of existing vessels permitting them to be more permissive to other angiogenic stimuli. Indeed, similar to VEGF, LRG1 can destabilize existing vessels and this may promote the angiogenic process. In addition, as LRG1 is ubiquitously present in the circulation it remains unclear what the relative contribution of systemic LRG1 vs. locally produced LRG1 is to vascular pathology. As with TGF-β, the effect of LRG1 on cell function is dependent on multiple factors one of which appears to be its local concentration, indicating a possible threshold effect achieved through local production. Moreover, the differential effect of luminal vs. abluminal exposure has yet to be determined as this may also dictate outcome.

**Table 1:** Evidence for the functional role of LRG1 in disease pathogenesis and vascular processes.

Disease/Condition	Process	Functional evidence	Publication
Cancer	Tumor progression, Angiogenesis (Bladder cancer)	Increased tumor cell proliferation, migration, invasion, EC angiogenesis (tube formation) (*in vitro*).	(80)
	Tumor progression, Angiogenesis (Pancreatic cancer)	Improved tumor cell viability, migration, invasion, EC angiogenesis (tube formation) through VEGFR activation (*in vitro*).	(81)
	Tumor progression, Angiogenesis (Gastric cancer)	Increased EC proliferation, migration, and angiogenesis (tube formation). Tumor cell angiogenesis and tumor progression (*in vitro*).	(27)
	Vessel normalization	Improved vessel structure and vascular function (Antibody blockade or gene deletion in tumor models *in vivo*).	(32)
	Metastasis	Promoted metastasis in lung cancer via NG2^+^ perivascular cells and STAT3 signaling (*in vivo*).	(29)
	Metastasis (Melanoma)	Reduced tumor cell metastasis, growth, proliferation, and angiogenesis in the absence of LRG1 (*in vivo*, *in vitro*).	(28)
	Angiogenesis (Non-small-cell lung cancer)	Promoted tumor cell proliferation, migration, invasion, EC angiogenesis (tube formation) through TGF-β (*in vitro*).	(82)
	Angiogenesis (Ovarian cancer)	Induced EC angiogenesis (tube formation) by upregulating VEGF, Ang1, TGF-β (*in vitro*).	(83)
	Angiogenesis (Colorectal cancer)	Promoted tumor cell invasion, EMT, EC migration, angiogenesis and sprouting (*ex vivo*, *in vitro*) through HIF-1α.	(84)
Diabetes	Angiogenesis	Induced angiogenic and neurotrophic function, EC angiogenesis (tube formation), proliferation, migration (*in vivo*, *ex vivo*, *in vitro*).	(85)
	Wound healing	Promoted EC viability, proliferation, migration, angiogenesis (*in vitro*), and wound healing (*in vivo*).	(86)
	Wound healing	Controlled immune cell infiltration, re-epithelialization, and EC angiogenesis/proliferation (tube formation) through phosphorylation of SMAD1/5.	(17)
	Wound healing	Corneal epithelial wound healing and nerve regeneration via regulation of matrix metalloproteinases (*in vivo*, *in vitro*).	(54)
	Angiogenesis	Induced angiogenesis (*in vivo*) through ALK1-SMAD1/5/8 in glomerular EC.	(53)
	Pathogenesis	Elevated expression in glomerular EC (*in vitro*).	(52)
	Angiogenesis	Elevated expression in glomerular EC and angiogenesis (tube formation) (*in vitro*).	(87)
Fibrosis	Skin	Induced EC proliferation, migration, and angiogenesis (*in vitro*) and promoted skin fibrosis through ELK1 and ERK signaling.	(41)
	Lung	Promoted lung fibrosis through TGF-β-induced Smad2 (*in vivo*, *in vitro*).	(42)
	Kidney, DKD	Induced expression in glomerular EC and promoted fibrosis through p38 signaling (*in vitro*).	(88)
	Kidney, CKD	Promoted EC angiogenesis (tube formation) and proliferation (protective role).	(34)
	Heart fibrosis, myocardial infarction	Gene ablation aggravated myocardial fibrosis and cardiac remodeling by suppressing SMAD1/5/8 (*in vivo*) (protective role).	(89)
Acute respiratory distress syndrome	Vascular repair, tissue healing	Exhibited angiogenic properties and tissue repair through TGF-βR2 and SMAD1/5/8.	(90)
Osteoarthritis	Angiogenesis	Induced EC angiogenesis and mesenchymal stem cell migration (*in vitro*).	(64)
Corneal neovascularization	Angiogenesis	Induced angiogenesis and lymphangiogenesis via activating VEGF signaling (*in vivo*).	(91)
Ischemia	Angiogenesis	Promoted blood vessel formation through upregulating the TGF-β1 signaling (*in vivo*).	(92)
Atherosclerosis	Calcification	Endothelial LRG1 induced VSMC de-differentiation and calcification through SMAD1/5 signaling (*in vivo*).	(93)
De novo angiogenesis	Vessel formation (placenta)	Exhibited pro-angiogenic functions and hypervascularization in gestational diabetic placenta (*ex vivo*, *in vitro*).	(58)
	Vessel formation	Induced *de novo* angiogenesis upon activation by IL-6/STAT3 (*ex vivo*, *in vitro*).	(26)
	Vessel formation (ocular)	Induced *de novo* angiogenesis through SMAD1/5/8 (*ex vivo*, *in vitro*).	(25)

EC, endothelial cell; EMT, epithelial to mesenchymal transition; CKD, chronic kidney disease; DKD, diabetic kidney disease; VSMC, vascular smooth muscle cells.

**Figure 3:**
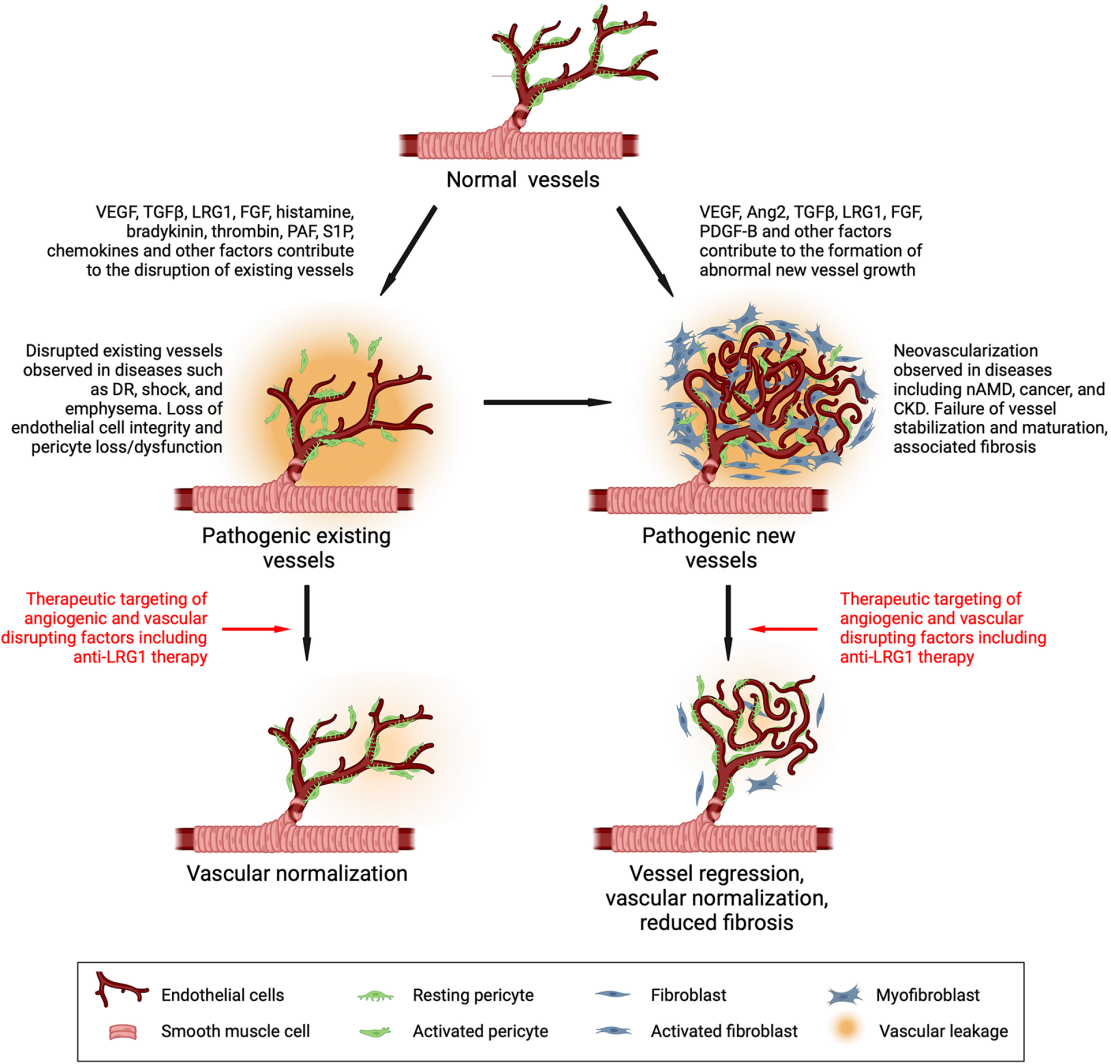
Drivers of vascular dysfunction and its therapeutic reversal. Vessels under normal conditions are stabilized and maintained in a mature functional state through cross-talk between the endothelial cell and mural cells (pericytes and smooth muscle cells). During disease, vasoactive factors are released that destabilize existing vessels and stimulate new dysfunctional vessel growth, both of which contribute to disease progression. Targeting factors that alter vascular function in disease is a common therapeutic objective. Inhibiting LRG1 activity has been shown to normalize vessels and may therefore be a promising therapeutic approach. DR, diabetic retinopathy; nAMD, neovascular age-related macular degeneration; CKD, chronic kidney disease. Created with BioRender.com.

The data above support the idea that in contrast to many angiogenic factors with homeostatic roles in the maintenance of a healthy vasculature, LRG1 expression is induced locally in response to pathogenic cues to drive the formation of a destabilized vasculature (Figure 3). These cues include inflammatory signals, hypoxia and vessel destabilizing factors that not only induce LRG1 expression but may also act on the vasculature independently creating an overall disruptive milieu. Acute inflammation, for example, causes dysfunction to existing vessels and often precedes neovascularization. Indeed, when inflammation becomes chronic and unresolved, it can promote the sustained activation of downstream mechanisms that trigger endothelial dysfunction, vascular remodeling, and abnormal neovascularization all of which serve as major risk factors for the development of cardiovascular disease (95). Our studies, and other recent evidence, demonstrate that endothelial cells themselves represent a key source of LRG1 in the diseased milieu (Figure 1). For instance, single cell RNA sequencing revealed *Lrg1* as one of the most upregulated genes in disease-associated endothelial cells in murine models of liver cancer (96) and atherosclerosis (97), while a seminal study demonstrated that LRG1 originating from endothelial cells contributes primarily, in an autocrine fashion, to vessel malfunction and disease severity in a model of emphysema (37). Interestingly, transcriptomics studies also showed higher *Lrg1* expression in endothelial cells isolated from LPS-treated lungs, pointing to inflammation-induced LRG1 being responsible for aberrant vasculogenesis (39). Similarly, *Lrg1* was found upregulated in diseased endothelium from inflamed retinal vessels in a mouse model of experimental autoimmune uveitis, suggesting a role for LRG1 in retinal inflammation and angiogenesis (98).

Abnormal angiogenesis is associated with numerous conditions, and the role of LRG1 in this pathology has become evident. Thus, as discussed in more detail below, it has been reported to be involved in neovascular dysfunction in ocular disease, diabetes, kidney disease, lung disease, impaired wound healing, inflammatory conditions, gestational diabetes and cancer. In addition, LRG1 may also contribute to destabilization of existing vessels by impacting on endothelial cell adhesion and permeability (Figure 3). Such emerging evidence highlights the potential of targeting LRG1 with novel therapeutic strategies to attenuate vascular disruption in disease. In support of this, and as highlighted elsewhere in this review, deletion of the *Lrg1* gene or blocking LRG1 function has been shown to partially reverse the vascular pathology in many conditions. Thus, therapeutic blockade of the angiopathic properties of LRG1 will therefore not only permit vessels to revascularize tissue in a more physiological manner but has the potential to re-establish a quiescent endothelial state in existing disrupted vessels.

**Figure 4:**
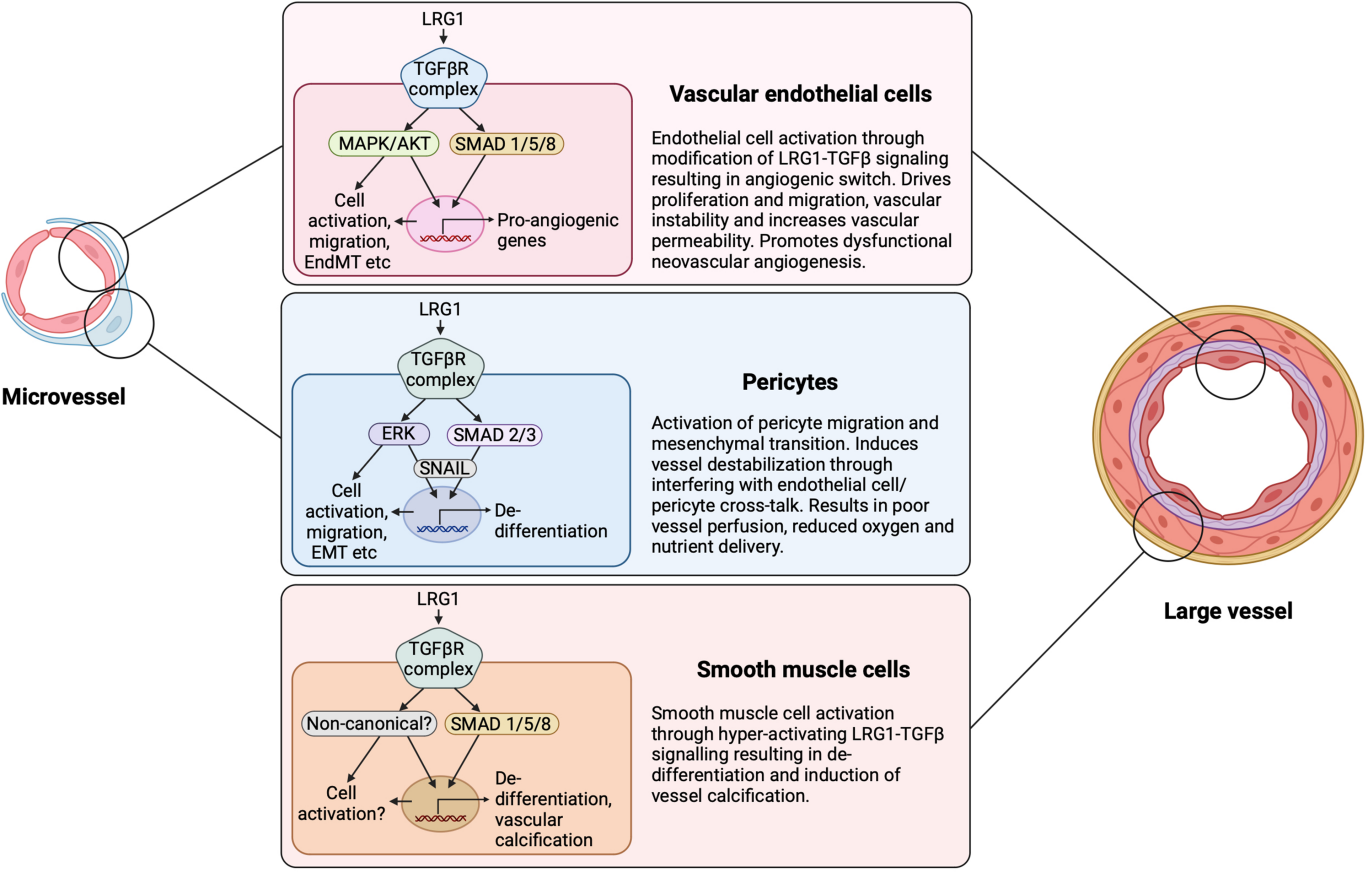
LRG1 cell-dependent signaling and outcome. LRG1 can induce signaling in all cellular constituents of the vasculature. Depending on the configuration of signaling receptors and downstream influences, LRG1 can cause the TGF-β angiogenic switch through acting on the endothelial cell, pericyte and smooth muscle cell. LRG1 has been shown to alter the canonical and non-canonical TGF-β signaling pathways in a cell-dependent manner. EndMT, endothelial to mesenchymal transition; EMT, epithelial to mesenchymal transition. Created with BioRender.com.

In order to devise therapeutic strategies targeting LRG1, it is important to understand the mode of action through which LRG1 promotes defective angiogenesis or impairs the established vasculature. To date, it is evident that TGF-β signaling is a key downstream mediator of LRG1 activity. In endothelial cells, TGF-β signals through binding to the TGF-βRII receptor followed by initiation of signaling either via the ALK1 or ALK5 kinase. In homeostasis, TGF-β signaling maintains endothelial cell quiescence by regulating ALK5 and the downstream SMAD2/3 transcription factors (99). However, high levels of LRG1 can shift the balance of endothelial TGF-β signaling towards the ALK1 kinase, which associates with Endoglin (ENG) to activate the pro-angiogenic SMAD1/5/8 arm of transcription factors leading to endothelial cell proliferation, migration and pathological angiogenesis (25, 26, 53, 99) (Figure 4). Indeed, ENG has been proposed as being essential to allow the interactions between LRG1, TGF-β and ALK1 (25, 100) but evidence from other cell types demonstrates that it can still elicit TGF-βRII signaling in the absence of ENG. Nevertheless, this so called TGFβ angiogenic switch is not a binary response as outcome is more likely due to the relative balance between these two canonical signaling arms that dictates the context-dependent nuanced response. Moreover, LRG1 also activates the non-canonical TGF-β pathway in various cells and settings (23), which may influence vascular structure and function (Figure 4). Merely promoting the pro-angiogenic TGF-β switch, therefore, is unlikely to fully explain the complex angiopathic effects of LRG1. There also exists the distinct possibility that LRG1 can modify BMP signaling although this remains to be determined. Outside the TGF-β axis, an alternative mechanism has been noted, whereby LRG1 binds to the Latrophilin-2 receptor to promote LRG1-dependent angioneurin effects in diabetes, where angiogenic and neurotrophic processes are in place (85). Additionally, recent studies have provided evidence that LRG1 disrupts vascular homeostasis by altering the fine balance between endothelial cells and pericytes causing leaky and destabilized vessels (26, 32). How this imbalance is achieved needs to be further explored, but autocrine and paracrine signaling mechanisms between the endothelium and pericytes appear to be critical. Those mechanisms may involve the release of angiocrine factors including Ang1 and Ang2, or LRG1-driven signaling in pericytes.

## LRG1, tissue repair and wound healing

Postnatally, angiogenesis is an important part of the repair and wound healing process. This involves a tightly regulated sequence of events including the recruitment of many different cell types driven in part by overlapping phases of inflammation and tissue remodeling (101). Failure of wound healing can lead to chronic wounds, which can be observed in many conditions such as eye disease, lung and heart disease, and diabetes (102). Upregulation of LRG1 in various cell types during the normal wound healing process clearly suggests its involvement, possibly through the promotion of new vessels and cell de-differentiation and migration (Figure 1). Whilst the role of LRG1 in normal blood vessel formation during wound healing is still unknown, reduced blood vessel density has been observed in the wound bed of *Lrg1*^−/−^ mice (17), suggesting its involvement in post-developmental vessel formation. In a murine skin wound healing model, LRG1 drives keratinocyte migration and re-epithelization by improving HIF-1α stability through ELK3 (103), whereas in a similar setting, LRG1 promoted endothelial cell proliferation, angiogenesis and EMT (17). In the latter study, LRG1 expression within the wound was also observed in bone-marrow-derived cells such as neutrophils, monocytes, macrophages and dendritic cells. The presence of LRG1 was shown to be essential for timely wound repair as *Lrg1^−/−^* mice exhibited a significant delay in wound closure, which was reversed when bone marrow-derived LRG1-expressing stem cells were transfused into the knock-out animals (17). However, in wounds from diabetic mice, as well as from patients with diabetes, LRG1 expression was markedly enhanced and in mice was shown to result in delayed wound closure. LRG1 was also associated with the adverse inflammatory response by stimulating excessive neutrophil attraction and promoting NETosis in a TGF-β/ALK5/ΑKT-dependent manner (17). The delay in wound healing in diabetic mice was reversed in *Lrg1^−/−^* mice, demonstrating its context dependent activity. In diabetic wounds in the rat, LRG1 has also been shown to increase blood vessel density through activating the WNT/β-catenin pathway, which, counterintuitively, may exacerbate the wound healing process (86).

Whilst not entirely consistent, these studies suggest that under normal conditions LRG1 promotes physiological tissue repair by promoting angiogenesis and cell migration, but in a chronic inflammatory setting, as seen in diabetes, LRG1 remains induced prolonging the initiating stage of wound healing (Figure 2). Our current understanding, therefore, is that LRG1 is induced as part of the acute innate immune response mechanism that sequesters Cyt c, and through modification of TGF-β signaling activates endothelial cells and pericytes resulting in destabilized vessels and stimulation of sprouting angiogenesis. We propose that through other switches in the TGF-β signaling, LRG1 also drives EMT that permits loss of epithelial junctional integrity, cell division and migration required for wound closure. As the inflammatory response resolves under normal conditions LRG1 expression is attenuated allowing for cellular re-differentiation, wound stabilization, and maturation. However, in the presence of a continuing chronic inflammatory stimulus LRG1 expression is sustained, and even increased, which maintains cells in a de-differentiated less mature state and thus prevents wound resolution and tissue homeostasis (Figure 2).

## LRG1, ischemia and stroke

A role for LRG1 has been reported to be part of the repair mechanism in cerebral damage due to ischaemic injury. Interestingly, high circulating LRG1 levels have been found positively associated with stroke severity and poor functional outcomes (104–106), as well as with poor cognitive impairment and neurological function (107). In the brain, following ischemic stroke injury a single cell transcriptomics study revealed the emergence of a LRG1-positive endothelial cell subpopulation in the brain infarct area, the expression profile of which suggests its involvement in the complex regulation of angiogenesis, with both pro- and anti-angiogenic factors expressed (108). In fact, in a mouse cerebral artery occlusion model, LRG1 increased apoptosis and autophagy through TGF-β and SMAD1/5 exacerbating the ischemic injury (109). Similarly, increased LRG1 was found in brain endothelial cells in a rat brain ischemia model with expression correlating positively with VEGF, Ang2 and TGF-β (92), indicating a protective role for LRG1 by promoting *de novo* formation of blood vessels, possibly through TGF-β (92). De novo vessel formation was confirmed, with CD34 staining showing significantly increased microvessel density after stroke, which again correlated with LRG1 expression (92). However, whilst new vessels were observed which may confer survival benefit, the likelihood is that these penumbral vessels are dysfunctional compromising their beneficial effect (110). A more recent study employing a cerebral ischemia-reperfusion injury model in the mouse demonstrated that in the *Lrg1*^−/−^ mouse there was reduced cerebral oedema and infarct size, which was accompanied by improvement in neurological function (111). Crucially, this study showed that following cerebral ischaemia, cells of the blood-brain barrier were able to retain the expression of barrier function related genes, such as claudin 11, integrin β5, protocadherin 9, and annexin A2, more effectively in the absence of LRG1 (111). This demonstrates that LRG1 has a detrimental effect on the blood-brain barrier and thus contributes to vasogenic cerebral oedema. In its wider role, *Lrg1* knockout also permitted a more anti-inflammatory and tissue-repairing environment and reduced neuronal cell death. In a different ischaemic setting, tissue remodeling after myocardial infarction involved the infiltration of LRG1 expressing myeloid cells that were reported to exert a cardioprotective role by promoting signaling through the pro-angiogenic TGF-β/SMAD1/5/8 axis and contributing to post-infarct vessel formation (89).

## LRG1 and vascular pathology of the eye

### Retinal neovascularization

Vascular problems of the eye, such as in diabetic retinopathy or neovascular (exudative or wet) age-related macular degeneration (nAMD), are characterized by vessel remodeling and angiogenesis and can occur intraretinally, subretinally or at the vitreal interface. Irrespective of the ocular disease, vascular dysfunction is often a major contributing factor to loss of vision in the developed world (112). The first evidence that LRG1 is actively implicated in pathogenic neovascularization emerged from its identification as the most up-regulated gene in a transcriptomics analysis of retinal microvessels isolated from three different animal models (retinal dystrophy 1, VLDLR knock out, *Grhl13^ct^/J* curly tail), each of which showed marked retinal vascular remodeling and pathology (25). This study went on to show that the *Lrg1* transcript is almost undetectable in the healthy mouse retina but in experimental retinal vascular diseases such as experimental choroidal neovascularization (CNV) (113) and oxygen-induced retinopathy (OIR) (114) that model choroidal and retinal neovascularization respectively, expression of LRG1 is induced in endothelial cells (25). In OIR, the abnormal retinal neovascularization that occurs at the vitreous interface is believed to be driven largely by hypoxia, whereas in CNV the laser burn to the retinal pigment epithelium (RPE) results in vessels sprouting from the choriocapillaris through the breached RPE into the subretinal space as a result of injury-induced hypoxic and inflammatory cues. Irrespective of the model system employed, genetic deletion of *Lrg1* led to a diminished pathogenic neovascular response coupled with a reduction in vascular leakage as assessed by fundus fluorescein angiography. Of note, however, revascularization of the hyperoxia-induced vessel ablated region in OIR proceeds in a more physiological patterned manner and is not affected by *Lrg1* deletion (25, 94), further demonstrating that LRG1 contributes to pathogenic, but not physiological, neovascularization. Similar findings were seen when LRG1 activity was blocked by intravitreal delivery of a specific function-blocking antibody, the application of which significantly reduced OIR and CNV lesion size (25, 94) and lends credence to its potential as a therapeutic target.

In addition to the disease-associated induction of endothelial LRG1 expression, myeloid cells have also been found to express LRG1 in choroidal neovascularization. A recent study exploring the transcriptional profiling of immunosenescence in myeloid cells during the development of CNV showed that aged senescent myeloid cells of CNV-induced animals exhibited an upregulated angiogenic transcriptional profile with significantly elevated *Lrg1* and *Arg1* expression compared to young cells (115). This is the first study to provide evidence of upregulated *Lrg1* expression in cells other than the endothelium contributing to choroidal neovascularization. Although it is not clear yet whether this is an epiphenomenon or there is a direct causal role for LRG1 in CNV-related immunosenescence, the data suggest a potential role for LRG1 and myeloid cells in the immunoregulation of CNV that merits further investigation.

Corroborating the mouse studies above, elevated expression of LRG1 has also been found in the aqueous and vitreous humour and in retinal choroidal neovascular membranes of nAMD patients (116–119), as well as in dry (atrophic) AMD (120). Mundo et al., showed that in ocular sections of nAMD membranes LRG1 is co-localized not only with endothelial cells but also with myofibroblasts, suggesting an alternative cell source through which LRG1 may exert its pathological effects (118). Thus, myofibroblast-derived LRG1 may contribute to endothelial dysfunction in the retina in a paracrine manner, but it may also be involved in promoting retinal fibrosis through autocrine mechanisms (118). This suggests that, two-way LRG1 cross talk between endothelial cells and fibroblasts may drive both vascular dysfunction and fibrosis. This is in accordance with other findings showing that LRG1 promotes fibrosis in various tissues and conditions including the skin (41), idiopathic pulmonary fibrosis (42), renal fibrosis (34) as well as diabetic nephropathy (33, 121), by directly affecting the physiology and activity of fibroblast-like tissue resident cells (Figure 1). Interestingly, a recent study showed that exogenous LRG1 promotes the EMT of RPE cells as evidenced through high levels of αSMA and fibronectin and low levels of ZO-1 (122). The concept that vascular dysfunction can contribute to fibrosis is not new (123) and it raises the interesting possibility that endothelial derived LRG1 may also trigger the fibrotic response in the eye. Similarly, endothelial cells have been shown to contribute to matrix deposition when they undergo phenotypic differentiation via EndMT, a process predominantly regulated by TGF-β. EndMT is a hallmark of cardiovascular disease and has been studied in a variety of experimental models (124). Although there is no known link between EndMT and LRG1, unpublished evidence from our laboratory suggests that LRG1 is overexpressed in a cytokine induced EndMT model system and may therefore play a role in the eye.

### Diabetic retinopathy

In diabetic retinopathy, where retinal vascular dysfunction, dropout and neovascularization are prominent, LRG1 has been reported to be upregulated not only in the vitreous humour and in ocular tissues (25, 50, 125, 126), but also systemically in the plasma of patients with proliferative diabetic retinopathy (PDR) (50, 127). Diabetic retinopathy is the most common complication of diabetes, leading to impaired vision and ultimately to vision loss (128). The first stage of the disease is non-proliferative and is characterized by microvascular abnormalities leading to macular oedema, microaneurysms, micro-haemorrhages, and poorly perfused, occluded, and de-endothelialized capillaries. In PDR, the later stage of the disease, loss of vessels and subsequent hypoxia results in the growth of abnormal neovessels leading to vitreous haemorrhage, tractional detachment and eventually blindness. Since LRG1 is almost undetectable in a healthy human eye, its elevated expression in the vitreous in diabetes is most likely to be due to activated local production in response to disease, but it may also derive from the plasma as a consequence of vascular leakage. Whether the upregulated expression drives the vascular complications in the eye or contributes to other diabetes-associated pathology remains to be elucidated. Indeed, accumulating evidence indicates that LRG1 is upregulated in the plasma (56, 129) and urine (130) of people with diabetes, hence it can serve as a biomarker for PDR (131). However, LRG1 did not offer a significant improvement when used in a risk prediction model for PDR (57). Nevertheless, the greater evidence so far implicates LRG1 in retinal neovascularization, with its genetic deletion or inhibition of function through antibody blockade ameliorating vascular pathology, lesion size and leakage in animal models (25, 94). In addition, unpublished data from our lab shows that LRG1 can promote the phenotypic and functional differentiation of pericytes towards a fibrotic state and that this contributes to vascular dysfunction in diabetic retinopathy through both canonical and non-canonical TGF-β signaling (20) (Figures 1, 4). This evidence highlights the possibility that endothelial derived LRG1 may also impact retinal function in a paracrine manner. Although our understanding of diabetic retinopathy has increased substantially, and anti-VEGF therapeutics have revolutionized diabetic macular oedema and PDR treatment, a substantial number of patients remain or become refractive which clearly implicates the involvement of other players and highlights the need for further research. The evidence to date suggests that LRG1 may be a contributing factor to resistance to anti-VEGF strategies and thus LRG1 blockade could improve outcome.

### Corneal neovascularization

The angiogenic properties of LRG1 have also been described in the context of corneal neovascularization in a corneal alkali burn mouse model, where LRG1 promoted a significant outgrowth not only of blood vessels, but also of lymphatic vessels (91). The normal cornea is avascular in order to maintain transparency but under pathological conditions, such as inflammation or trauma, new vessels invade the avascular tissue causing visual impairment. In *Lrg1* knock down studies using siRNA, a limited angiogenic and lymphangiogenic response was observed compared to control mice (91). In this study, different members of the VEGF family were implicated in LRG1-driven corneal angiogenesis and lymphangiogenesis, with VEGF-A/B and their receptors VEGFR-1/2 regulating the former and VEGF-C/D together with VEGFR-2/3 regulating the latter (91). How LRG1 impacts on this remains unclear but it raises the possibility of cross regulation between these pathways. Apart from neovascularization, LRG1 was also found to promote corneal fibrosis by increased deposition of αSMA, collagen type I and CTGF in the corneal epithelium (48). This LRG1-driven effect was mediated by neutrophil infiltration at the site of corneal injury through the phosphorylation of STAT3 and the upregulation of IL-6/STAT3 signaling (48). These data point to LRG1 as a potential therapeutic target to ameliorate pathogenesis in corneal disease.

## LRG1 and chronic kidney disease

The role of LRG1 in kidney disease, in particular diabetic nephropathy, is gaining interest (132) with growing evidence that LRG1 contributes to vascular rarefaction and abnormal neovascularization. In normal kidney, LRG1 expression is found in glomerular endothelial cells co-localized with that of CD31, as well as in the tubulointerstitium (53). The kidney is a highly vascularized organ, where maintenance of normal blood flow is crucial for renal function which, following pathological complications such as vessel loss and fibrosis, may become seriously compromised. Endothelial dysfunction coupled with abnormal angiogenesis have long been known to contribute to the pathogenesis of diabetic nephropathy and other chronic kidney conditions, although the underlying molecular mechanisms are poorly understood (33, 53, 133). Several studies show that in a variety of chronic kidney disease (CKD) models LRG1 gene and protein expression is significantly increased. Indeed, a transcriptomics study has shown that in a model of diabetic nephropathy, *Lrg1* was upregulated in glomerular endothelial cells, where it mediated high glucose-induced pathological angiogenesis (87). Similarly, other studies have also shown that in experimental diabetic kidney disease (DKD) *Lrg1* gene expression induced in glomerular endothelial cells is involved in vascular rarefication and subsequent neovascularization and fibrosis, partly via activation of the p38 and TGF-β-SMAD1/5/8 pathway (52, 53, 87, 88). Consistent with these reports, LRG1 overexpression has also been shown to result in exacerbation of disease (33, 53). What is interesting, but needs further corroboration, is the suggestion that the initiating mechanism driving vascular dysfunction is mediated by LRG1 and is independent of VEGF. This is based on the finding that LRG1 expression precedes the expression of VEGF and its receptor VEGFR-2 in diabetic nephropathy (52) and that VEGF is expressed mainly in the injured podocytes following endothelial injury (134, 135). These data prompted the authors to suggest that glomerular endothelial LRG1 may be an initiating factor in vascular pathology in the diabetic kidney.

In various other *in vivo* models of CKD, including the albumin overload model (136), unilateral ureteral obstruction (UUO) model (34), and the aristolochic acid-induced nephrotoxicity (AAN) model (33), LRG1 protein levels have also been shown to be higher, often correlating with increased pro-inflammatory and pro-fibrotic cytokines. Such findings support the contention that LRG1 is not only induced by inflammatory cues but that it also promotes the renal inflammatory response including the activation of macrophages in a TGF-βR1-dependent manner (137).

In the kidney, endothelial cells are not the only source of LRG1, as it has also been shown that renal tubular epithelial cells (33) and HK-2 human proximal tubular epithelial cells (34) can be induced *in vitro* to express LRG1. Tubular epithelial cell derived LRG1 has been shown to activate the TGF-β-SMAD3 pathway in fibroblasts resulting in increased renal fibrosis (33). This additional cellular source of LRG1 may not only trigger fibrosis but also contribute further to endothelial cell dysfunction. Indeed, it has also been proposed that LRG1 mediated microvascular dysfunction in the kidney may facilitate the onset and progression of fibrosis (34), supporting the notion that these two phenomena are interrelated (123). What is clear from these studies is that in the kidney LRG1 not only stimulates vessel loss and the formation of abnormal neovessels, it also drives the fibrotic response, all of which combine to reduce glomerular filtration rates (121, 138). In accordance with the studies above, increased renal LRG1 expression is reflected in the urine, where significantly elevated levels correlate closely with the degree of renal tubular dysfunction (136). Consistent with these findings, *Lrg1* knockout reduces the deterioration of kidney function (33, 53). Similarly, suppression of LRG1 and ALK1-dependent angiogenesis by metformin showed significant renoprotective effects in a diabetic rat model (139).

Clinical evidence also points to LRG1 playing a role in the pathogenesis of CKD. Thus, it has been shown that in people with type 2 diabetes and DKD, plasma LRG1 levels predict both albuminuria and CKD progression beyond traditional risk factors (56, 140). Similarly, in children with type 1 diabetes a clear relationship between plasma LRG1 and estimated glomerular filtration rate (eGFR) decline suggests that LRG1 may be an early marker of DKD progression (49). In separate studies, using human DKD tissue, *LRG1* gene expression has been found to be increased in glomerular endothelial cells (53). Moreover, in other forms of CKD, including lupus nephritis (36), IgA nephropathy (34), and end-stage kidney disease dialysis patients (35), increased LRG1 plasma and biopsy tissue levels correlate with worse outcome, increased inflammatory markers, and greater fibrosis. Of note, LRG1 levels correlated positively with IL-6, a known activator of LRG1 gene expression, as well as with a more advanced state of T cell differentiation and the presence of cardiovascular disease and peripheral arterial occlusive disease (35) demonstrating its potential systemic involvement.

In addition to plasma, increased urine LRG1 levels in diabetes is also associated with an increased risk of progression to end stage kidney disease independent of traditional cardiorenal risk factors (55), and in kidney transplant recipients LRG1 has been considered a potential kidney injury marker that correlates with other tubular injury markers and functional deterioration (141). These human data support the evidence from experimental studies that LRG1 is an important factor in driving CKD through initial effects on the kidney vasculature and the subsequent fibrotic response. One of the most compelling pieces of clinical evidence that LRG1 contributes to DKD, however, was a recently reported GWAS study in people with type 2 diabetes and CKD, where a 5'UTR variant (rs4806985) in the promoter region of *LRG1* was found to influence its gene expression resulting in elevated plasma LRG1 and a robust association with increased risk of rapid decline in kidney function (51). This is the first study describing a polymorphism risk to LRG1 circulating levels, suggesting a potential use for LRG1 in stratifying patients with diabetes into subsets based on their genetic predisposition. Additionally, genetically influenced plasma LRG1 levels were also associated with lower cognitive function, further supporting a role for LRG1 as a novel biomarker for cognitive decline in type 2 diabetes mellitus (142). The conclusion drawn in many of these studies is that LRG1 is a potentially important therapeutic target as it is seen as a master upstream orchestrator of pathogenic TGF-β signaling. It is well established that the TGF-β pathway has a critical role in neovascular and fibrotic processes and that targeting constituents of this pathway continues to be considered an attractive therapeutic strategy. However, the need to retain homeostatic TGF-β signaling remains a challenge but one which may be overcome by targeting LRG1 as this is a key upstream factor in causing the switch from quiescent housekeeping to pathogenic disruptive signaling.

## LRG1 and lung disease

Inflammation, tissue repair, endothelial dysfunction and increased interstitial pressure are common phenomena in pulmonary disease leading to prominent vascular-related complications such as pulmonary embolism, abnormal microthrombi, and microvascular damage (143). Alveolar epithelial and endothelial permeability are also compromised with impaired gas exchange and vascular leakage due to loss or destabilization of cell junctions. In addition, the damaged endothelium may disrupt vascular tone and cause dysregulation of anti-inflammatory and anti-thrombogenic endothelial properties, and together with damaged epithelium can trigger the tissue repair process (144). To date, evidence shows that LRG1 is involved in pulmonary vascular dysfunction with increased expression seen in lung disease, including chronic obstructive pulmonary disease (COPD), interstitial pneumonia, airway inflammation in asthma, and active tuberculosis, with LRG1 levels serving as a biomarker for early diagnosis, progression, and prognosis (38, 66, 145–148).

Several reports show that LRG1 is upregulated in lung epithelial and endothelial cells, mainly but not exclusively in inflammation-induced pathology (37, 39, 66, 90). In particular, in human COPD tissue, upregulated LRG1 was localized specifically to the endothelium and correlated positively with marked airflow obstruction, decline in lung function and severity of emphysema (37). COPD is a heterogeneous long term lung disease characterized by persistent airway inflammation, microvascular dysfunction, dysregulated angiogenesis, and endothelial apoptosis (149). Specific endothelial deletion of LRG1 in a murine elastase model of COPD protected against severe parenchymal destruction, highlighting a critical role for LRG1 in promoting the development of maladaptive lung vasculature (37). Although the exact mechanism that mediates this process has not yet been defined, it is possible that LRG1 impacts angiogenic responses following endothelial injury either by promoting defective angiogenesis or through the development of fibrosis. Indeed, LRG1 has been shown to trigger a pro-fibrotic response in the lung by activating lung fibroblasts and the subsequent production and deposition of extracellular matrix via TGF-β signaling and the phosphorylation of SMAD2 and SMAD3 in mouse bleomycin models (42, 44). Strikingly, in a recent single cell transcriptomics study, *LRG1* was described as an extracellular matrix coding gene and its expression was found increased and maintained at high levels in aging lung, which associated LRG1 with age-related inflammation and tissue stiffness (150). However, LRG1-mediated fibroblast activation and proliferation was not regulated by SMAD2 or 3, the levels of which were either unchanged or repressed, respectively, implying that other signaling mechanisms, such as activation of alternative canonical or non-canonical pathways, are in place (150). Indeed, consistent with previously published data (25, 26, 53), in viral-induced lung injury, LRG1 upregulation drives endothelial cell proliferation and the angiogenic responses required for tissue repair by activating SMAD1/5 signaling (90).

Elevated levels of circulating LRG1 have also been reported in severe COVID-19 patients in blood, plasma, and tissue proteomic studies (151–156). Although most of these studies associate elevated LRG1 expression with an early immune and inflammatory response, it is possible that LRG1 exerts an angiopathic role in the pulmonary microvasculature related to COVID-19. In fact, a dysregulated cytokine immune response, known as a “cytokine storm”, has been established and studied extensively in patients with COVID-19 (157). This includes highly elevated expression of pro-inflammatory cytokines that correlates with COVID-19 disease severity and requires immediate attention, as excessive activation of immune cells can lead to complicated and potentially lethal medical syndromes (158). Among others, IL-6 is a key player in this cytokine response with significantly elevated circulating levels in the plasma of patients with COVID-19, and consequently it has been reported to contribute to the related vascular pathology (159, 160). Blocking IL-6 signaling as a therapeutic intervention has been extensively studied in many diseases (161, 162), and COVID-19 randomized controlled clinical trials with biologics targeting the IL-6 receptor, including the Tocilizumab antibody, have shown evidence of clinical benefit (163–166). Elevation of both IL-6 and LRG1 in patients with COVID-19 suggests that circulating pro-inflammatory IL-6 may induce systemic and local upregulation of angiopathic LRG1 in the pulmonary microvasculature. Indeed, IL-6 upregulates LRG1 expression in human pulmonary microvascular endothelial cells, and this effect is reversed when IL-6 signaling is blocked by the tocilizumab antibody (26). Upregulated LRG1 in turn may contribute to the development of a destabilized vasculature with prominent endothelial dysfunction and vascular leakage during impaired lung tissue wound healing (Figures 1, 4). This LRG1-dependent microangiopathy may be mediated by the pro-angiogenic TGF-β-SMAD1/5 signaling arm, as evidenced in other endothelial-related disease settings (25, 26, 90). As reported in experimental CKD, and described above, dysregulated microvascular endothelial cells in the lung in COVID may also be a driver of fibrosis (37), linking LRG1 to these two key pathogenic processes.

## LRG1 and inflammation-associated disease

Over the past decade several studies have provided evidence that LRG1 is involved in various inflammatory and autoimmune diseases, and may act as a useful clinical and diagnostic biomarker (23, 167–174). In such conditions, levels of LRG1 are upregulated at the site of inflammation, with expression induced by pro-inflammatory cytokines secreted by various cell types, followed by the initiation of a series of downstream vascular events. During inflammation, one of the key vascular responses is an increase in vessel permeability that, alongside other inflammatory changes, facilitates the extravasation of immune molecules and cells to the site of injury (175). In addition to tissue resident cells, recruited immune cells provide an additional source of angiogenic factors that play a part in the inflammatory and reparative response. Over time this response resolves but under certain chronic conditions unresolved inflammation, including the persistence of LRG1 expression, continues through an imbalance of stimulatory, inhibitory and disruptive factors, and can give rise to long-term vasculopathic outcomes (176) (Figure 2).

Osteoarthritis (OA) is the most common inflammatory joint disorder, with aberrant endothelial cell proliferation, vascular penetration, and synovial fibrosis being the main disease leading mechanisms that contribute to structural damage and pain (177). *Lrg1* transcript has been found to be upregulated in chondrocytes upon IL-6 stimulation (178). In OA the pro-inflammatory cytokine TNF-α, a key player in the pathophysiology of the disease and major activator of pro-angiogenic factors (179, 180), was also shown to induce LRG1 expression in the subchondral bone and articular cartilage (64). In this setting it promoted angiogenesis, mesenchymal stem cell migration and aberrant bone formation via MAPK-dependent p38/p65 signaling. This highlights a new potential mechanism through which LRG1 promotes abnormal neovascularization coupled with *de novo* bone formation. Supplementary to its role in angiogenesis in OA, LRG1 has also been shown to contribute to synovial fibrosis and joint stiffness by promoting secretion of extracellular matrix in synovial cells, cell migration and wound healing (46), further suggesting that LRG1 not only affects endothelial cells but also other cells in the cartilage exerting multiple parallel pathogenic responses.

Although LRG1 has attracted substantial interest with regards to its use as a clinical biomarker in a plethora of inflammatory conditions, little is known about its mechanistic involvement in the development and progression of vascular defects in these conditions. Nevertheless, LRG1 has been implicated in Kawasaki disease, an acute systemic vasculitis causing inflammation of small to medium sized blood vessels resulting in cardiovascular complications (181, 182). The main cytokines responsible for inducing LRG1, TFN-α and IL-6, are elevated in the plasma of patients with Kawasaki disease, and a proteomic analysis of serum exosomes of patients with coronary artery aneurysms caused by Kawasaki disease showed upregulated LRG1 levels, although no causal link with pathology was shown (183). Similarly, another study on Kawasaki disease in children identified LRG1 as a potential trigger of endothelial cell activation and cardiac remodeling that closely associated with IL-1β signaling (184). The cell source contributing to increased circulating LRG1 levels in these conditions has not yet been identified but evidence from other conditions suggests that upon inflammatory stimulation LRG1 is most likely produced by endothelial cells, where it exerts its vasculopathic effects via autocrine and paracrine pathways on the endothelium and the adjacent mural cells, respectively. Although the exact vascular pathogenesis in Kawasaki disease is not well understood, vascular complications include necrotizing arteritis associated with neutrophilic and immune cell infiltration, the release of pro-inflammatory cytokines, luminal myofibroblast proliferation and progressive obstruction of the coronary lumen have all been linked with LRG1 in other conditions and so it is likely that it plays a role in this and other vasculitides. In support of this, LRG1 has also been found to be a promising serum biomarker for large vessel vasculitis (LVV) (185), and antineutrophil cytoplasmic antibody (ANCA)-associated vasculitis (AAV) (186, 187).

Inflammation is a hallmark of cardiovascular disease and destabilized vasculature, and frequently serves as a trigger during the early stages of disease, while increased expression of inflammatory cytokines is associated with a higher risk of cardiovascular diseases (188). Thus, LRG1 may be anticipated to initiate or mediate inflammatory responses to some extent in conditions with cardiovascular risk. In fact, LRG1 has been shown to promote cardiovascular disease by regulating endothelial dysfunction and inflammation through TGF-β and SMAD1/5/8 signaling in endothelial cells, interrupting normal endothelium-dependent vasodilation and availability of nitric oxide (129). Furthermore, in a recent study employing the Western diet apolipoprotein E knockout (*ApoE*^−/−^) mouse model of atherosclerosis, LRG1 was detected within the atherosclerotic plaque, particularly in calcified regions (93). The cell source of LRG1 was found to be endothelial cells that had been activated by inflammatory mediators. Furthermore, this study demonstrated that LRG1 was responsible for inducing vascular smooth muscle cell activation and vessel calcification via a SMAD1/5 signaling pathway (Figure 4). The authors conclude that LRG1 is a significant contributor to the development of plaque complications and therefore a potential therapeutic target. On the other hand, and in contrast to most other studies, in arterial stenosis it was suggested that endothelial LRG1 could serve as a negative regulator of inflammation in response to TNF-α by inhibiting expression of ICAM1 and VCAM1 and thus blocking monocyte recruitment, offering a significant atheroprotective effect (189). Interestingly, this is consistent with what has been observed in cancer (see below) where endothelial anergy, as indicated by ICAM1 and VCAM1 expression, is reversed upon LRG1 inhibition.

Exactly how LRG1 mediates vascular inflammation is not clear although there is growing evidence for LRG1 regulating a pro-inflammatory accumulation of immune cells. In particular, LRG1 is involved in neutrophil function modulating NETosis, which is closely associated with inflammatory processes (17). NETosis has been described as a form of necrosis and is associated with many diseases including COVID, thrombosis and CKD, as well as wound healing and other vascular processes (190–192). As mentioned above, a recent study suggested that LRG1 mediates NETosis and contributes to poor wound healing in diabetic mice (17). Although future work is required to unravel the role of LRG1 in regulating neutrophil function, we speculate that NETosis might be an important additional mechanism through which LRG1 may impact on vascular structure and function.

## LRG1 and cancer

LRG1 expression has been studied in a range of malignancies where it has been shown to be elevated and to associate with poor prognosis and survival (23, 61, 62, 193, 194). In addition, raised blood LRG1 levels have been established as a tumor biomarker with potential clinical value and also as a predictive marker for cancer onset (30, 59, 60, 62, 193, 195–200). A growing number of studies support an integral role for LRG1 in cancer, where it has been shown to control cell viability and apoptosis, and promote epithelial cells to undergo EMT, a crucial step in tumor progression and metastasis (27, 28, 30, 31, 80–84, 201–203)*.* In particular, LRG1 acts directly on tumor cell proliferation, migration, and invasion contributing to tumor growth and survival (Table 1), and these functions have been described in detail elsewhere (23, 29, 193). Consistent with LRG1 playing a role in tumor progression, LRG1 blockade in different tumor models inhibits growth and improves survival and thus has been proposed as a potentially beneficial therapeutic target (29, 32). There is also accumulating evidence that LRG1 has important angiocrine and angiopathic functions in cancer, not only by promoting the development of destabilized and immature neo-vessels, but also by impairing already established co-opted vasculature (Figure 3). As widely acknowledged, growing tumors require a constant supply of oxygen and nutrients, and serving these needs often relies on a concomitant developing vascular network. The tumor vasculature, however, is typically abnormal exhibiting impaired structure and function. In particular, tumor blood vessels are immature, tortuous and chaotic in organisation, with an abnormal vessel wall characterized by a discontinuous endothelium, incomplete coverage of mural cells, and atypical basement membrane structures leading to poor perfusion and leakiness (204). These characteristics also create a hypoxic and acidic environment within the tumor tissue that favours malignancy and metastasis and combine to reduce immune cell infiltration and effective immune responses, and restrict the delivery of therapeutics and effectiveness of radiotherapy.

The angiogenic potential of LRG1 has been assessed and described in various types of tumors, including colorectal, gastric, pancreatic, ovarian, and non-small-cell lung cancer (193). Specifically, LRG1 has been proposed to enhance the angiogenic process through acting directly on endothelial cells to induce proliferation and migration, but also indirectly through stimulating proangiogenic factors such as VEGFA (84). Moreover, LRG1 has been associated with increased microvessel density, suggesting that it impacts tumor vascular growth (26). As in other diseases, the direct effect of LRG1 on vessel function is believed to be mediated primarily through modification of canonical TGF-β and SMAD signaling, but in all likelihood also through hyperactivation of non-canonical TGF-β pathways. Alternative mechanisms for LRG1-driven angiogenesis have been proposed including regulation through HIF-1α, which is associated with resistance to cancer chemotherapy and increased patient mortality (205). HIF-1α knockdown was shown to block LRG1-mediated angiogenesis, EMT, and tumor invasiveness, and is consistent with LRG1 being induced in response to hypoxia (84). In another study, ERK mediated phosphorylation of ELK4 in a human colorectal cell line resulted in complex formation with SP1/3 and the induction of *LRG1* gene expression (69). This, it was argued, results in enhanced tumor angiogenesis through activation of the TGF-β-SMAD1/5 pathway in endothelial cells. Whatever the mechanism, the evidence is clear that LRG1 plays a central role in driving abnormal vessel formation in solid tumors.

In line with LRG1 driving vessel abnormalization, strong evidence indicates that vessel structure and function in tumors can be improved by knocking out *Lrg1* or by its inhibition. Indeed, restoring vessel function, a process referred to as vascular normalization, represents a promising strategy to facilitate drug delivery, enhance cytotoxic T cell function, and increase the tumor response to standards of care and immunotherapies (206, 207). In this context, *Lrg1* gene deletion, or functional blockade of the protein, has been shown to improve tumor vascular function as manifested by better perfusion, reduced tumor hypoxia and reduced vascular leakage (32). In particular, vessel size, basement membrane and perivascular mural cell coverage of the endothelium were all significantly increased in the absence of LRG1 (32). As a likely consequence of vascular normalization, LRG1 inhibition not only led to significant improvements in the delivery and efficacy of anti-tumor therapies, but also improved immune-cell infiltration (32). This may partly be explained by re-activation of anergic endothelial cells to allow leukocyte infiltration, seen for example by increased ICAM-1 and VCAM-1 expression. Collectively, these data show that the angiopathic functions of LRG1 not only promote a pro-oncogenic vascular microenvironment in primary and metastatic tumors, but also contribute to immune modulation. Unpublished data from our lab show that in addition to the vascular normalization effects, inhibition of LRG1 also promotes tumor infiltration of T-cells by modulating the immunosuppressive tumor microenvironment, thereby supporting a switch from being immunologically “cold” to “hot”.

Angiogenesis is not the only means through which tumors obtain a vascular supply. Vessel co-option is a surrogate mechanism whereby tumor cells employ the established vasculature to support growth, survival, and metastasis. In a recent study where, in the presence of sunitinib, tumor growth escapes from VEGF-dependent angiogenesis through vessel co-option, single cell transcriptomics revealed a surprisingly similar signature between tumor co-opted endothelial cells and pericytes and their healthy non-tumor bearing counterparts (208). This finding was further confirmed in other vessel co-opted tumor metastatic models. The similarity in the cell transcriptome was predominantly due to the lack of genes associated with angiogenesis and pericyte activation that are observed in angiogenic tumors. Intriguingly, however, *Lrg1* was found to be one of the top 10 genes that were differentially expressed in co-opted postcapillary vein endothelial cells compared to normal endothelium (208). This suggests that *Lrg1* is one of the few genes to be expressed in both tumor angiogenic and co-opted endothelial cells. Further studies, however, will be required to determine whether *Lrg1* induction in co-opted vessels exerts similar vasculopathic effects as observed in angiogenic tumor vessels and in vessels of other diseases.

Aside from endothelial cells, a major cell source of LRG1 in cancer is frequently the tumor cells (Figure 1) but this is not always the case. As in other diseases, there is evidence that LRG1 is also expressed by other cell types including fibroblasts, and immune cells. In all experimental studies conducted thus far, however, LRG1 expression has been shown to co-localize with vessel markers, such as CD31 and CD34 (60, 209) illustrating its ubiquitous presence in tumor endothelial cells. Recent work on the role of LRG1 in cancer showed that in some cancer models *Lrg1* expression was mostly restricted to the vascular endothelium, with no expression detected in the perivascular mural cell population or the cancer cells themselves (29, 32). Nevertheless, this was sufficient to impact on tumor growth as *Lrg1* knock-out or antibody blockade were still effective in reducing tumor growth. Interestingly, using similar tumor models it has been shown that the primary tumor induces systemic vascular LRG1 expression and that this primes the vascular metastatic niche and promotes tumor metastasis (29). This priming was also associated with an expansion of NG2^+^ perivascular mural cells, which have been described as effective mediators of metastasis (210). LRG1 induction in the tumor mass and systemically in cancer is most probably through IL-6 and STAT3, with contributions from other signaling pathways. Indeed, in metastasis models the STAT3 signaling pathway has been shown to mediate LRG1-driven tumor metastasis, and that this can be significantly reduced in *Lrg1* deficient mice or following LRG1 antibody blockade (28, 29, 31). Through a different mechanism, liver endothelium-derived LRG1 has been shown to promote tumor growth and metastasis of colorectal cancer in a paracrine manner through binding to the HER3 receptor, leading to its phosphorylation and activation (202). If evidence from other diseases translates to cancer, other cell sources of LRG1 are likely to impact the tumor microenvironment. In particular, fibroblasts and neutrophils are a key source in other disease settings and their contribution to cancer merits further investigation.

## Conclusion

Over the last 10 years our understanding of how the secreted glycoprotein LRG1 contributes to physiological and pathological processes has grown exponentially and, in the latter case, demonstrates beyond doubt that it plays a significant contributing role in disease. Whilst we are only just beginning to appreciate the extensive biological role of LRG1, it is clear that much of its activity is mediated through its switching effect on the ubiquitous and complex TGF-β signaling network. Not surprisingly, therefore, the biological effect of LRG1 is wide ranging and highly cell and context dependent, reflecting the biological diversity of TGF-β activity. Accordingly, LRG1 exerts pleiotropic effects depending on the cell target and the influence of other environmental cues, affecting not only the vasculature but also other cell types that are under the influence of TGF-β including epithelial cells, cancer cells, immune cells, and fibroblasts, that in turn may also feedback to affect vascular function. Whilst much speculation remains surrounding the normal physiological role of LRG1, the weight of evidence that it exerts pathological effects is now compelling even though its acceptance as a mainstream pathogenic effector molecule is only just gaining traction. Amongst its effects, those it has on the vasculature are likely to be of substantial clinical importance in a wide range of diseases including cancer, chronic kidney disease, diabetic retinopathy, and emphysema. Indeed, its potential role as a major pathogenic mediator of systemic cardiovascular disease is only just beginning to be considered.

LRG1 has been found to be over-expressed in many disease tissues, where its local production, especially under chronic inflammatory conditions, appears to exacerbate pathological cell dysfunction. This is in line with its likely physiological role as a component of the repair mechanism. Thus, LRG1 can induce de-differentiation of epithelial, endothelial and pericyte cells to support the wound healing process, but under chronically stressed cell conditions, LRG1 is not switched off and its persistence has destabilizing effects resulting in aberrant pathological responses (Figure 2). In the context of vascular function, LRG1 can act on endothelial cells and mural cells affecting the fine interactive balance needed for a stable and mature vasculature (Figure 4). In its sustained and heightened presence, existing and new vessels become unstable and reactive resulting in vascular leakage, fragility, and the failure of new vessels to mature (Figure 3). It presents, therefore, an intriguing and potentially valuable therapeutic target. Critically, LRG1 is particularly attractive in the context of therapeutic targeting of TGF-β signaling as this has been fraught with setbacks, predominantly because TGF-β and its receptors all have critical housekeeping roles. To date no essential homeostatic role for LRG1 has been described rendering it a potentially more suitable therapeutic target. Thus, we reason that inhibiting LRG1 will block the pathogenic activity of TGF-β without disturbing these key homeostatic functions. We anticipate, therefore, that over the next decade our understanding of LRG1 biology will be substantially enhanced and that its therapeutic targeting in multiple indications will be well advanced.
